# Systematic evaluation of plant metals/metalloids accumulation efficiency: a global synthesis of bioaccumulation and translocation factors

**DOI:** 10.3389/fpls.2025.1602951

**Published:** 2025-06-05

**Authors:** Wenbin Huang, Chengnian Zhang, Bowei Zhu, Xiaoying Liu, Huxuan Xiao, Shibin Liu, Huaiyong Shao

**Affiliations:** ^1^ College of Ecology and Environment, Chengdu University of Technology, Chengdu, Sichuan, China; ^2^ College of Earth and Planetary Sciences, Chengdu University of Technology, Chengdu, Sichuan, China; ^3^ The 4th Geological Brigade of Jiangxi Geological Bureau, Nanchang, Jiangxi, China; ^4^ The 4th Geological Brigade of Sichuan, Chengdu, Sichuan, China; ^5^ State Key Laboratory of Geohazard Prevention and Geoenvironment Protection, Chengdu University of Technology, Chengdu, Sichuan, China; ^6^ College of Geography and Planning, Chengdu University of Technology, Chengdu, Sichuan, China

**Keywords:** hyperaccumulator, soils, metals/metalloids, bioaccumulation, translocation, phytoremediation

## Abstract

Phytoremediation, which involves the use of plants to accumulate and translocate metals and metalloids, represents a promising strategy for environmental remediation. The efficiency of phytoremediation is influenced by many factors such as metal/metalloid types, soil properties, and plant traits. It remains unclear how these factors modulate the efficiency of phytoremediation. We synthesized 547 data pairs from 82 studies to comprehensively evaluate the ability of hyperaccumulating plants to accumulate and translocate metals/metalloids under varying environmental conditions. The results show that cadmium (Cd), the most frequently investigated heavy metal, has the highest average bioaccumulation factor (BF) (10.0 ± 1.3) but a relatively low average translocation factor (TF) (1.8 ± 0.1). Aboveground biomass (AGB) of Cd hyperaccumulators is negatively correlated with BF but positively correlated with TF. Cd hyperaccumulating plants exhibit the highest accumulation capacity (maximal BF = 191), with roots outperforming aerial parts. The lower TF is mainly due to the lower AGB of Cd hyperaccumulating plants. In contrast, nickel (Ni) hyperaccumulators exhibit the highest TF, particularly in leaves and stems, indicating that Ni primarily accumulates in the aboveground parts. As soil pH increases, the BF of Cd and Zinc (Zn) decrease, whereas the BF of lead (Pb) increases, likely due to their distinct chemical behaviors under different pH conditions. Threshold concentrations were also identified for several for metals/metalloids (e.g., Cd: 214.8 mg kg^-1^; Pb: 31352.3 mg kg^-1^), beyond which BF falls below 1.0, indicating diminished accumulation efficiency due to toxicity constraints. In sum, these findings provide insights for optimizing phytoremediation strategies, aiding in plant selection and remediation condition optimization for improved efficiency and sustainability.

## 1 Introduction

Soils are a fundamental component of both ecosystems and human well-being, providing food, fiber, and biodiversity protection (Amundson et al., 2015). However, rapid industrialization has led to widespread contamination of soils by toxic metals and metalloids, posing serious environmental and health risks (Khalid et al., 2017). Over 10 million sites globally are contaminated, covering around 80,000 km² (Kumar et al., 2019). These metals are toxic and bioaccumulate, threatening human health and food security (Song et al., 2021). Although various remediation technologies are available, many are cost-intensive and technically complex, limiting their practical application. In this context, phytoremediation—recognized as a low-cost, environmentally friendly strategy—has emerged as a promising alternative (Rajendran et al., 2022). By leveraging the natural ability of plants to extract, stabilize, or degrade contaminants, phytoremediation offers significant potential for the remediation of metal/metalloid-contaminated soils (Ehsan et al., 2014).

Phytoremediation, defined as the use of plants to extract, immobilize, contain, or degrade contaminants from soil, water, or air (Gerhardt et al., 2017). Its effectiveness depends largely on selecting plant species that can both adapt well to local climatic conditions and tolerate metal toxicity while accumulating high levels of metals and metalloids (Fernández et al., 2017). Such plants, commonly referred to as hyperaccumulators (Brooks et al., 1977), are characterized by their exceptional ability to concentrate specific metals or metalloids in their shoots above a given environmental threshold (e.g., lead (Cd) > 100 mg kg^-^¹ dry weight, Zinc (Zn) > 10,000 mg kg^-^¹ dry weight) (Kramer, 2010; Pollard et al., 2014). Despite high contaminant levels, these plants can successfully complete their life cycle. The capacities of hyperaccumulators to accumulate and translocate metals/metalloids are key determinants of phytoremediation potential (Galal and Shehata, 2015). These capacities are commonly quantified using the bioaccumulation factor (BF) and the translocation factor (TF). Although there are several definitions, BF is most frequently calculated as the ratio of metals/metalloids concentration in the plant’s shoot to that in the soil, while TF represents the ratio of metal concentration in the shoot to that in the root (Buscaroli, 2017). Numerous studies have proposed criteria for evaluating hyperaccumulation based on these factors, with BF > 1 and TF > 1, along with metal concentrations in the shoot exceeding threshold values, commonly accepted as indicators of hyperaccumulation (Li et al., 2018).

Although extensive investigations have been conducted on hyperaccumulators, the mechanisms underlying substantial variations in BF and TF across plant species remain poorly characterized. For instance, under identical conditions, Cd accumulation in *Noccaea caerulescens* can vary by up to sevenfold among different populations (Roosens et al., 2003). Moreover, BF and TF values vary significantly across different metals and plant taxa. Among known hyperaccumulators, approximately 400 species from 40 families are capable of accumulating Ni (Jaffré et al., 2013), whereas Pb hyperaccumulators are extremely rare, with only around 14 species identified (Tang et al., 2009). A notable example is *Oryza sativa* (rice), which accumulates elevated levels of arsenic (As) under flooded conditions but shows limited uptake of Cd, Pb, and chromium (Cr) in its shoots (Wan et al., 2019). These observations underscore the pronounced metal- and species-specific patterns of hyperaccumulation and reinforce the importance of further research to clarify its physiological and environmental determinants, as addressed in this study.

In addition to intrinsic plant traits, climatic characteristics, soil properties, and experimental conditions significantly affect the accumulation and translocation capacities of hyperaccumulators (Oyuela Leguizamo et al., 2017). Increased precipitation, for instance, has been associated with elevated As concentrations in *Pteris vittata*, potentially due to enhanced soil moisture that stimulates plant growth (Marchiol et al., 2004; Zeng et al., 2022). Soil pH also plays a critical role, influencing the bioavailability of metals/metalloids through changes in the exchange capacity between metals/metalloids cations and H^+^ ions adsorbed on soil particle surfaces (Fayiga and Saha, 2016). When pH decreases, metals/metalloid ions desorb from colloidal and clay mineral surfaces, enter the soil solution, and thereby enhance metals/metalloids uptake by plants. This mechanism may explain the observed link between rhizosphere acidification—triggered by elevated phosphorus levels—and increased manganese (Mn) hyperaccumulation (Degroote et al., 2018). Although numerous studies have examined individual factors affecting metal uptake and translocation in hyperaccumulators, most have focused on isolated variables (Yanai et al., 2006; Jacobs et al., 2019). A comprehensive, quantitative understanding of how these factors collectively regulate the phytoremediation efficiency of metals/metalloids is still lacking.

Here, we compiled 547 data pairs from 82 peer-reviewed studies, which were identified by a systematic search of the ISI Web of Science and China National Knowledge Infrastructure Database (CNKI) platforms until September 2023, using terms related to hyperaccumulation and target metals/metalloids (e.g., Zn, As, Cd, Cr, Cu). Average BF and TF for different metals/metalloids were calculated. The key factors influencing phytoremediation, such as soil properties, plant species, climatic characteristics, and experimental conditions, were identified, and their relationships with BF and TF were analyzed. This study aims to: (1) systematically evaluate plant capacity for accumulation and translocation of various metals and metalloids (e.g., Cd, Ni, copper (Cu), Mn, Zn, Pb, As, iron (Fe)) by analyzing BF and TF across different species and experimental conditions; and (2) to determine the influence of key environmental variables, including soil properties (such as pH and total metals/metalloids content), experimental setups, and climatic characteristics, on the BF and TF values, with a particular focus on three ecologically significant elements: Cd, Pb, and Zn. The findings will provide critical insights into the dominant factors governing the hyperaccumulation of metals/metalloids in plants, thereby offering technical assistance for advancing the phytoremediation of metals/metalloids contamination.

## 2 Materials and methods

### 2.1 Data collection and extraction

A comprehensive literature search was performed using the ISI Web of Science and CNKI platforms to identify relevant primary studies published prior to September 2023. The search terms included (“hyperaccumulation” or “hyperaccumulation plant”) combined with (“Heavy metal” or “Metalloid*” or “Zinc” or “Arsenic” or “Cadmium” or “Chromium” or “Copper”). Studies meeting the following criteria were included in the database: (i) the article explicitly stated, or experimentally confirmed, that the plant species is a hyperaccumulator of metals/metalloids; (ii) no exogenous substances were added during the experiment that could affect the bioaccumulation and translocation of metals/metalloids in plants; (iii) at least one of the factors, BF or TF, was reported; and (iv) BF was calculated as the ratio of metals/metalloids content in shoot-to-soil, and TF was calculated as the ratio of metals/metalloids content in shoot-to-root.

A total of 82 studies, comprising 547 observations, met the inclusion criteria and were integrated into the database for further analysis. In addition to BF and TF, we also extracted data related to plant characteristics, native soil properties, climatic characteristics, and experimental conditions. Plant characteristics included aboveground and belowground biomass, as well as the concentrations of metals/metalloids in various plant organs (e.g., roots, stems, leaves, and shoots). Original soil properties encompassed soil organic carbon (SOC), total nitrogen (TN), available nitrogen (AN), available phosphorus (AP), and pH. All data on organic carbon and soil nutrient content were recorded in mg kg^-^¹ or g kg^-^¹. In addition, we collected data on total and available metals/metalloids concentrations in soils. When soil organic matter content was provided, it was converted to SOC using a conversion factor of 1.724 (Brooks et al., 1977).

For climate data, mean annual temperature (MAT) and mean annual precipitation (MAP) were extracted exclusively from field studies to evaluate the effects of temperature and precipitation on plant metals/metalloids accumulation and translocation capacities. For studies lacking direct climate data, MAT and MAP were obtained from WorldClim v2.1 (https://worldclim.org/data/worldclim21.html) based on reported coordinates using ArcGIS 10 software (Hijmans et al., 2005). Studies conducted under natural conditions were classified as “Field”, whereas those performed in greenhouses or pots were categorized as “Lab”. Additionally, studies were designated as “Historically” if this study indicated that soils had been naturally contaminated with metals/metalloids over a period, while studies where metals/metalloids were artificially introduced into soils were assigned to the “Newly” group.

### 2.2 Data statistics and analysis

The mean and standard error (SE) of BF and TF were calculated for the metals/metalloids. Statistical differences in BF or TF between “Field” and “Lab” groups, as well as between “Historically” and “Newly” groups, were tested using paired sample t-tests (*p* < 0.05). For data that did not follow a normal distribution, non-parametric tests were applied to assess the significance of differences in BF or TF between these groups. Exponential or linear regression analyses were conducted to examine the relationships between BF or TF and soil pH, as well as total or bioavailable metals/metalloids concentrations. Specifically for Cd—the largest dataset available—Spearman’s rank correlation was used to examine the relationships of BF and TF with factors such as climate, original soil properties, experiment duration, and plant biomass. Additionally, for Cd, we calculated BF for different hyperaccumulator plant organs (roots, stems, and leaves) and employed a binomial method for model fitting.

Egger’s regression test was performed to assess potential publication bias using the log-transformed BF and TF values collected from the 82 studies. If neither SD nor SE was available, the SD was estimated as 10% of the corresponding mean value (Liu et al., 2025). A funnel plot was also used to visually inspect the symmetry of the value distribution. Leave-one-out sensitivity analysis was conducted to evaluate the robustness of the pooled estimates; each observation was sequentially excluded, and the mean value recalculated. Ninety-five percent confidence intervals (CIs) were computed for each leave-one-out estimate. Linear mixed-effects model (LMM) was used to examine the interaction effects of environmental factors on metal uptake with the lmerTest package in R software for significance testing. The response variable was the log-transformed BF. Soil pH, total metal/metalloid content, and their interaction were modeled as fixed effects, while plant species was included as a random intercept to account for interspecific variability. All continuous predictors were standardized prior to model fitting to facilitate comparison of regression coefficients on a common scale. Model assumptions (e.g., normality, linearity, homoscedasticity, and multicollinearity) were checked using the performance package. Interaction effects were visualized using the effects package, and plots were customized with ggplot2 in R software (Bates et al., 2015).

## 3 Results

### 3.1 Mean and ranges of BF and TF

Funnel plots combined with Egger’s regression test were used to evaluate potential publication bias. For BF, the regression intercept was 3.501 (*p* = 0.050, *R* ^2^ = 0.340), suggesting marginally significant asymmetry. However, the Trim-and-Fill method did not impute any missing studies, indicating that although small-study effects may exist, their impact on the pooled estimates is likely minimal, and the overall results remain robust (Sterne and Egger, 2001) (**Supplementary Figure S1**). In contrast, the funnel plot for TF showed a more balanced distribution. Egger’s regression yielded an intercept of -2.402 (*p* = 0.139, *R* ^2^ = 0.445), indicating no statistically significant publication bias for TF. Leave-one-out sensitivity analysis indicated that the pooled mean remained stable across iterations (Δ < 0.015), with all estimates falling within narrow 95% confidence intervals (**Supplementary Figure S3**), further supporting the robustness of the findings.

**Figure 1:**
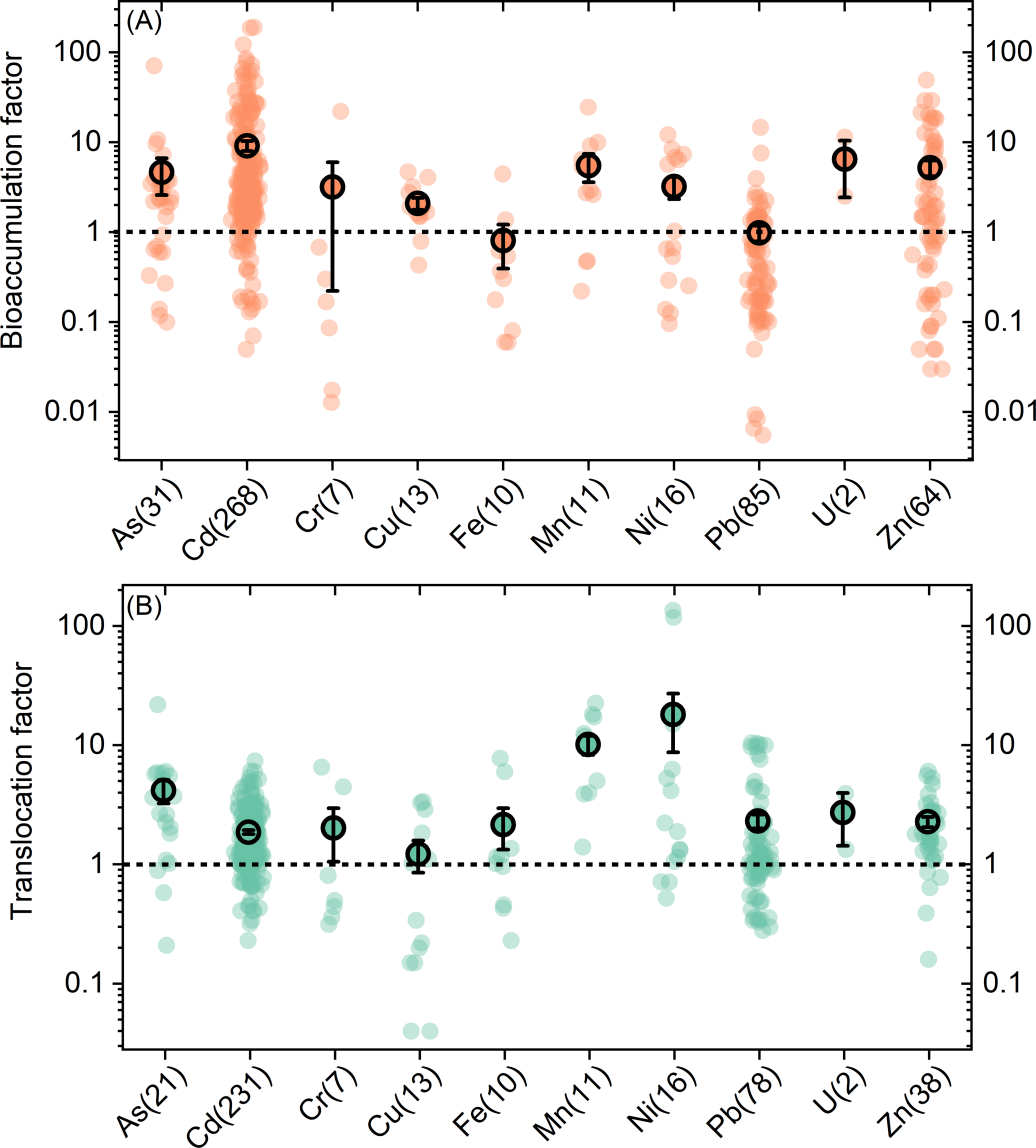
Bioaccumulation **(A)** and translocation **(B)** factors for different metals/metalloids. The numbers in the brackets are the observation numbers included in this study. The black circle points and error bars represent the average values and standard errors.

The ability of plants to accumulate and translocate metals/metalloids can be evaluated by BF and TF, respectively (**Figure 1**). For Cd, BF ranges from 0.1 to 191.0, with an average of 10. The average BFs for Mn, Zn, and As were 5.9 ± 2.1, 5.6 ± 1.1, and 4.9 ± 2.3, respectively. Plants’ abilities to accumulate Ni, Cu, and Cr are comparable, average BFs of which were 3.4 ± 0.9, 2.2 ± 0.3, and 3.3 ± 3.3, respectively. Furthermore, the average BFs for Pb and Fe are relatively low, at 0.9 ± 0.2 and 0.8 ± 0.4, respectively.

The average TF for all metals/metalloids in the database is greater than 1.0. Among them, Ni has the highest average TF (19.7 ± 10.6), followed by Mn (10.6 ± 2.0) and As (4.2 ± 0.9). Interestingly, although Cd has the highest average BF, its average TF is relatively low, at only 1.8 ± 0.1. Cu demonstrated the weakest translocation capacity among all elements, with an average TF of 1.1 ± 0.4.

### 3.2 Impact of experimental conditions on BF and TF of plants

**Figure 2:**
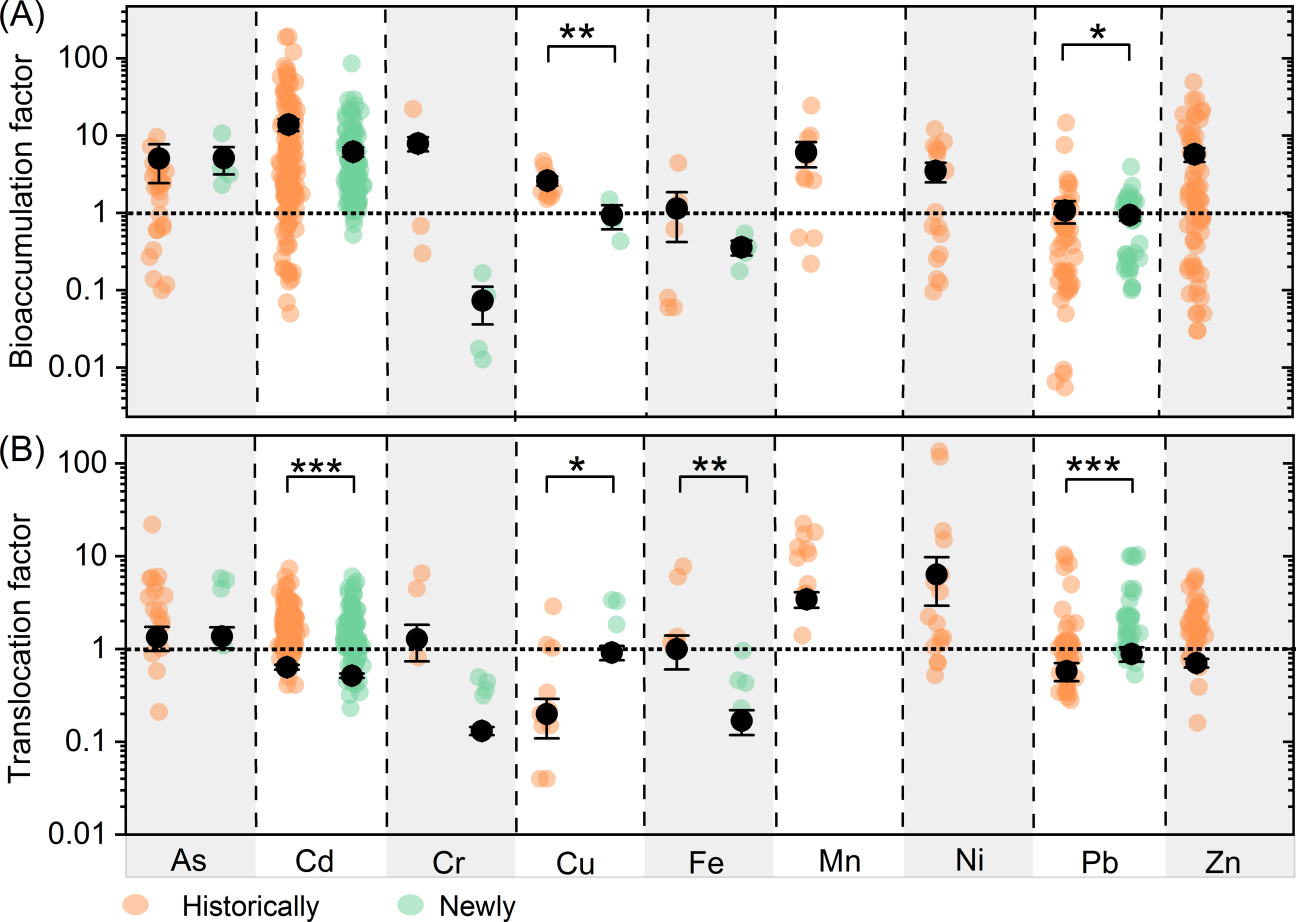
Comparisons of bioaccumulation **(A)** and translocation **(B)** factors between historically and newly contaminated observations. Different colors represents significant difference between historically and newly contaminated observations. Scatter plots show the distribution of bioaccumulation and translocation factors, respectively. ***, **, * represent p<0.001, p<0.01 and p<0.05, respectively.

Plants’ abilities to accumulate Cu and Pb (i.e., BF for Cu and Pb) are stronger in historically contaminated soils than in newly contaminated soils; however, their abilities to translocate Cu and Pb (i.e., TF for Cu and Pb) are weaker in historically contaminated soils (**Figure 2A**). Additionally, the average TFs for Cd and Fe are higher in historically contaminated soils compared to newly contaminated ones, while the average BFs for these metals remain similar between the two types of soil contamination (**Figure 2B**).

**Figure 3:**
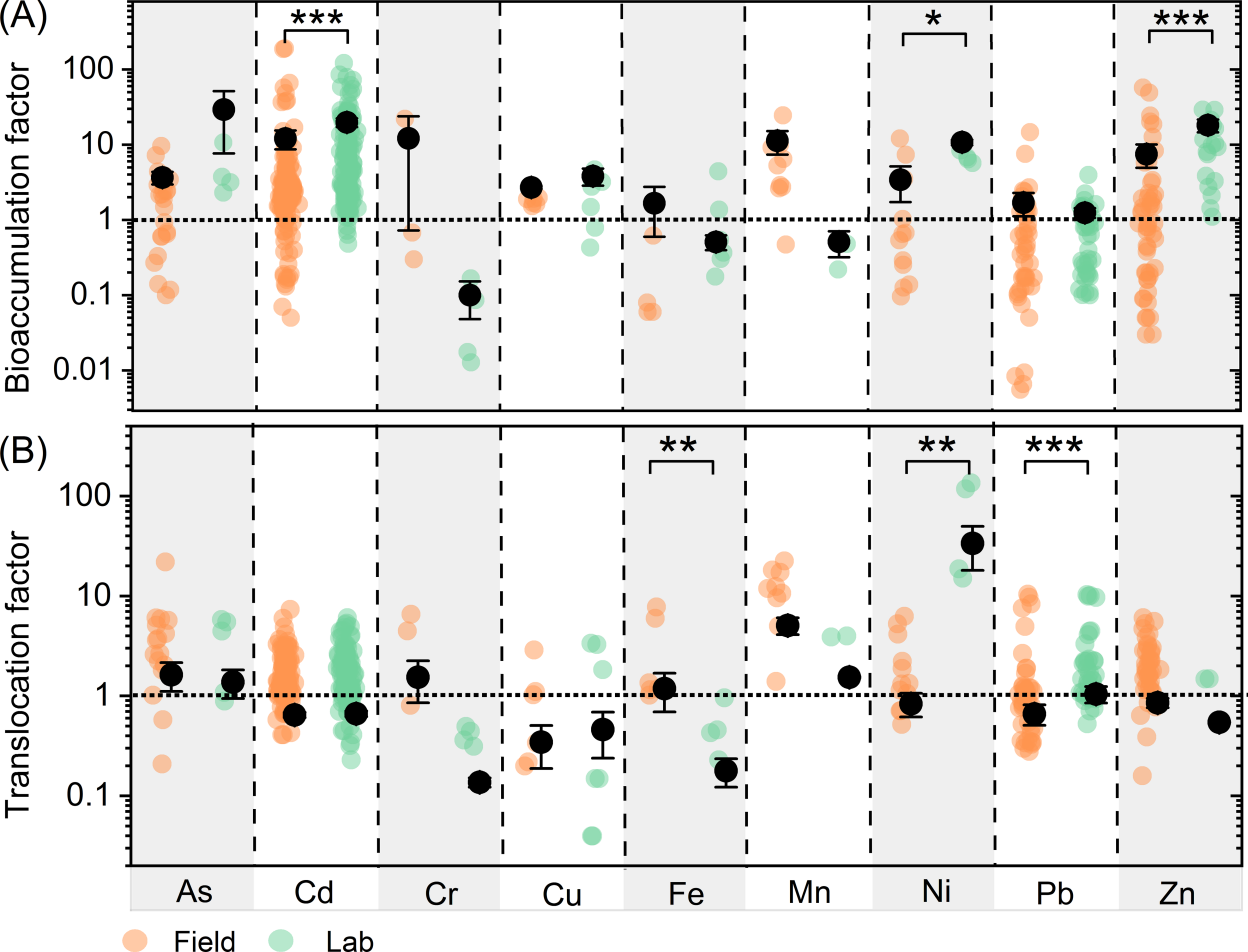
Comparison of bioaccumulation **(A)** and translocation **(B)** factors between field and laboratory studies of different metals/metalloids. Different colors letters represent significant differences between field studies and laboratory studies. Scatter plots show the distribution of bioaccumulation and translocation factors, respectively. ***, **, * represent p<0.001, p<0.01 and p<0.05, respectively.

The average BFs for Cd, Ni, and Zn are higher under lab conditions than under field conditions (**Figure 3A**). Similarly, the average TFs for Ni and Pb are greater under lab conditions compared to field conditions (**Figure 3B**). In contrast, plants exhibit a stronger ability to translocate Fe under field conditions than under lab conditions.

### 3.3 Bioaccumulation factor as affected by soil pH, total and available metals/metalloids content

**Figure 4:**
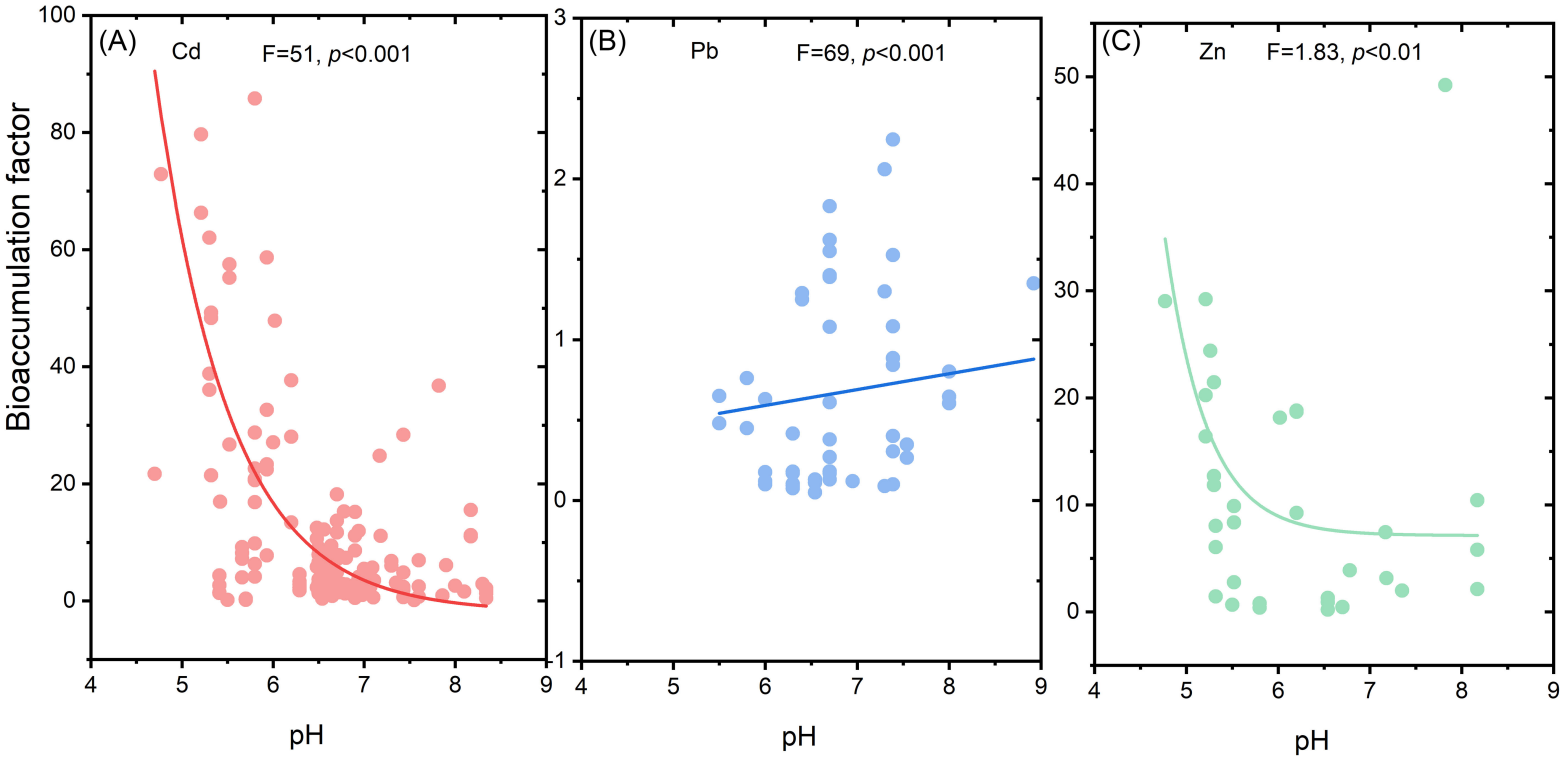
Relationships between bioaccumulation factor and soil pH. Subplots represent relationships between BF of Cd **(A)**, Pb **(B)**, Zn **(C)** and soil pH. Power and linear regression model were used to correlate the relationships between BF and soil pH. *p* value shows the significance of the regression.

Based on the database size, we analyzed the relationships between soil pH and BFs for Cd, Pb, and Zn (**Figure 4**). Exponential regression analysis revealed that BFs for Cd (**Figure 4A**) and Zn (**Figure 4C**) decreased gradually with increasing soil pH. In contrast, BF for Pb showed a positive correlation with soil pH (**Figure 4B**).

**Figure 5:**
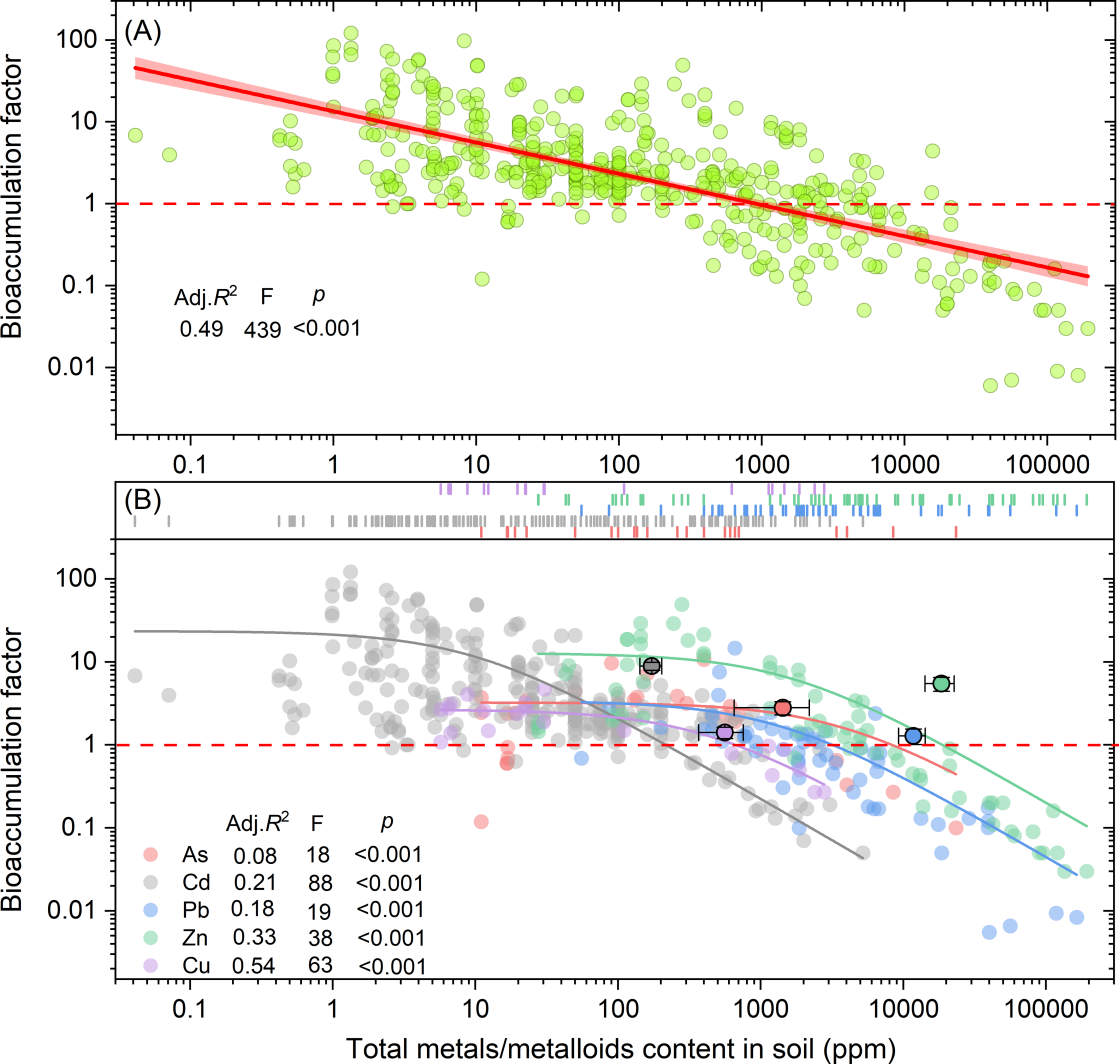
Relationship between bioaccumulation factor and total metals/metalloids content **(A)** in soil. The dash lines mark the total metals/metalloids content in soil when BF is equal to 1.0. Adj. *R* ^2^, F value and *p* value show the regression results for Cd, As, Pb, Cu and Zn **(B)**, respectively.

To further elucidate the relationship between total metals/metalloids content and the BF of hyperaccumulators, linear regression analysis was applied (**Figure 5A**). The results indicated a negative correlation between BF and total metals/metalloids content (*p* < 0.001, *Adj. R²* = 0.49). Thresholds for total metals/metalloids content to determine whether BF values exceed or fall below 1.0 were also identified. These threshold values were 214.8, 669.5, 31352.3, 8291.1, and 18657.3 mg kg^-^¹ for Cd, Cu, Pb, As, and Zn, respectively (**Figure 5B**).

**Figure 6:**
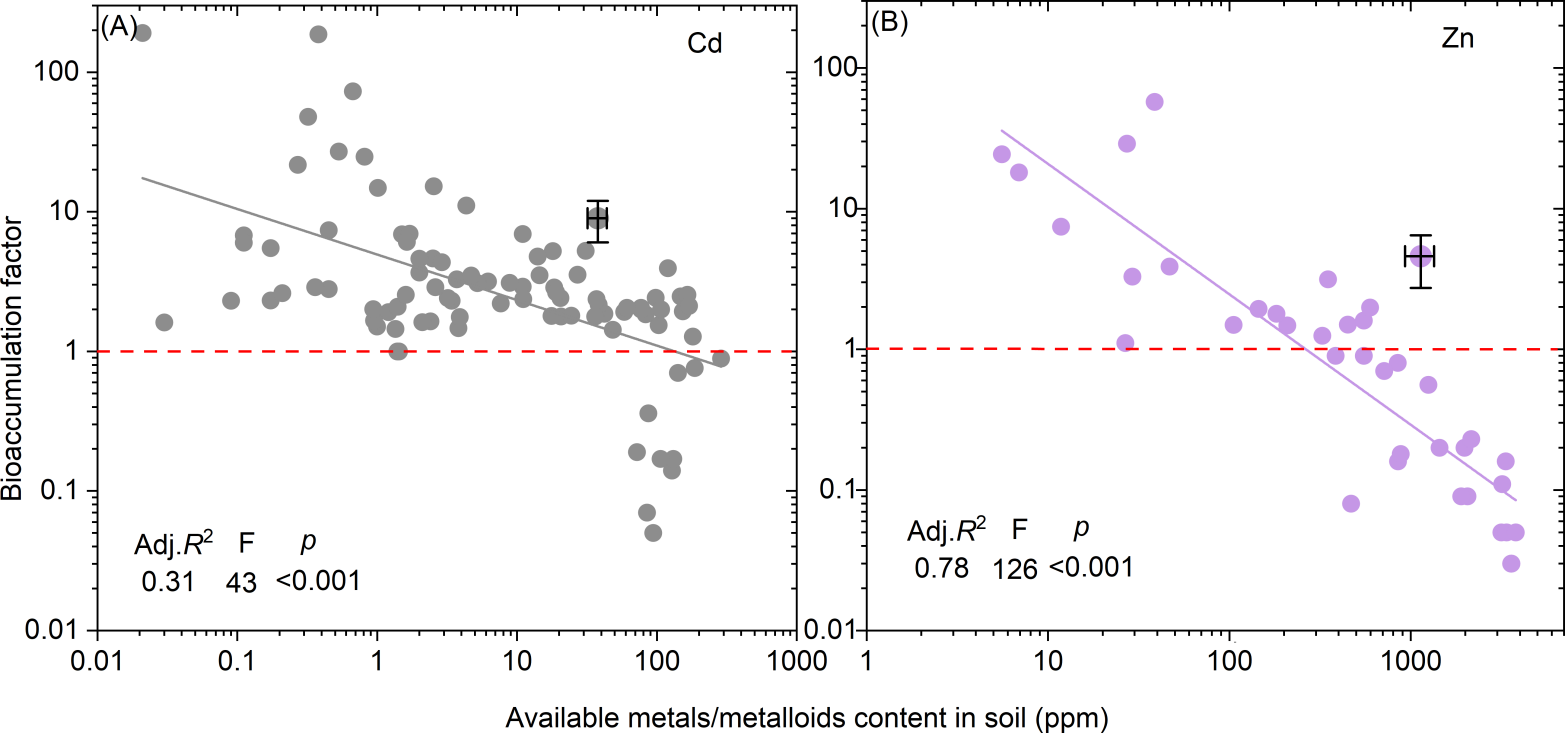
Relationship between available metals/metalloids content in soil and bioaccumulation factor. Based on the database, only the relationships for Cd **(A)** and Zn **(B)** are developed by a linear model. *p <*0.001 represents significance of the regression. The available contents of Cd and Zn when BF=1.0 are marked on the figures.

The relationships between the BFs of Cd and Zn and their available contents were analyzed and established through a linear regression model (**Figure 6A, B**). The results demonstrated that BFs for both Cd and Zn decreased as available Cd and Zn contents increased. The thresholds for available Cd and Zn content to determine BF values above or below 1.0 were identified as 131.7 mg kg^-^¹ for Cd and 260.1 mg kg^-^¹ for Zn.

### 3.4 BF and TF for Cd as influenced by climatic characteristics, experimental duration, soil properties and plant characteristics

**Figure 7:**
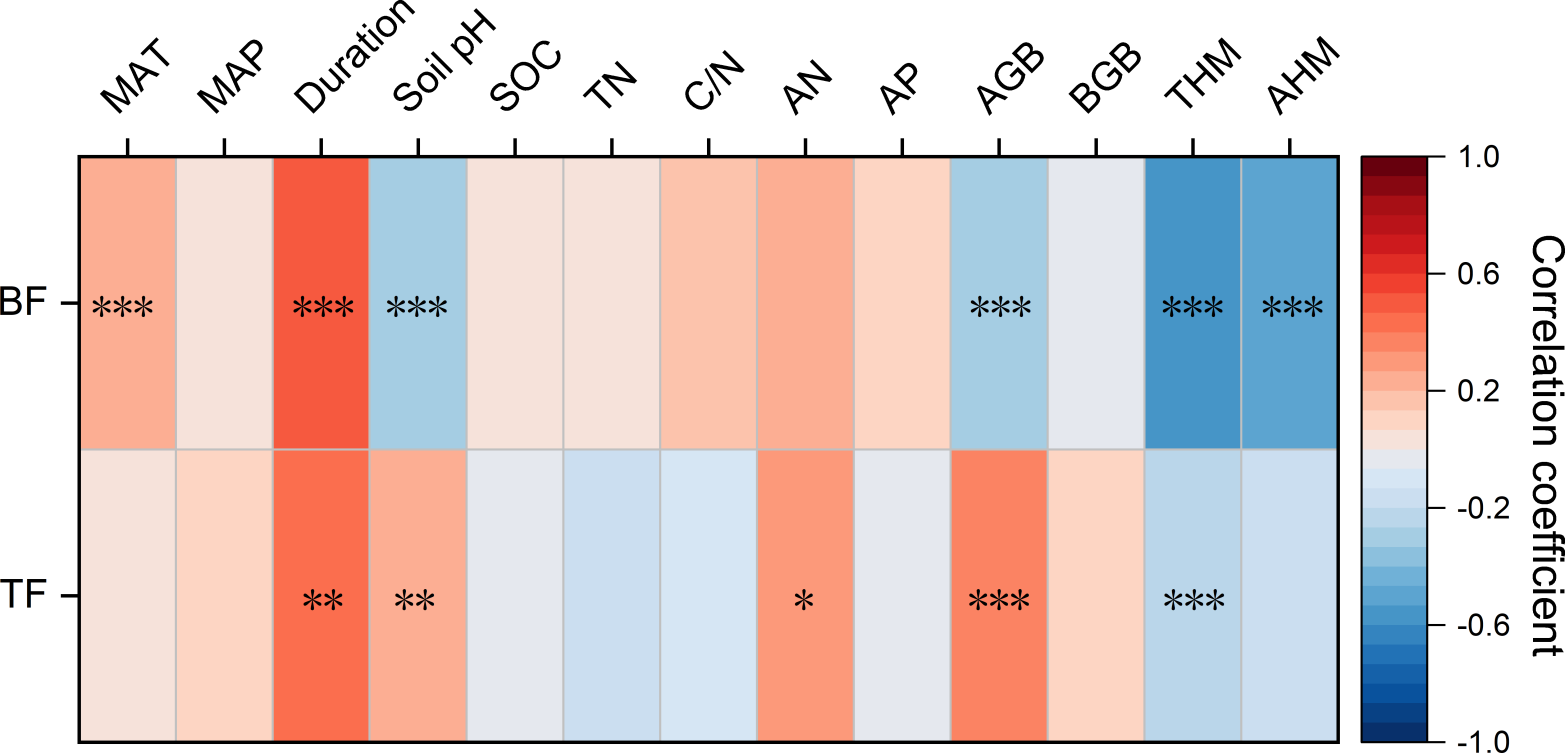
Spearman’s ranking correlation between bioaccumulation factor and translocation factor and climate, experiment duration, soil properties and plant characteristics for Cd. Color band shows the correlation coefficient. Asterisks show the significance of the correlation. ***, **, * represent *p <*0.001, *p <*0.01 and *p <*0.05, respectively.

Spearman’s rank correlation was applied to examine the relationships between BF or TF and various influencing factors, including climatic characteristics, experiment duration, soil properties, and plant characteristics, to assess their effects on the plant’s capacity to accumulate and translocate Cd (**Figure 7**). The results indicated a significant positive correlation between BF and MAT (*p* < 0.001). Both BF and TF increased with longer experimental duration. Above ground biomass (AGB) was negatively correlated with BF but positively correlated with TF (*p* < 0.001). Additionally, TF showed positive correlations with soil pH and AN, while exhibiting a negative correlation with total Cd content.

A significant interaction was found between soil pH and total metals/metalloids content (*p* < 0.05), suggesting that the effect of pH on metals/metalloids uptake varies with contamination level. All key model assumptions—including linearity, homoscedasticity, normality of residuals, and low multicollinearity—were verified using the performance package (see **Supplementary Figure S4** for diagnostic plots). As illustrated in **Supplementary Figure S4**, at lower metals/metalloids concentrations (3.39 mg/kg), increasing soil pH led to a greater reduction in log(BF), indicating inhibited bioaccumulating capacity. In contrast, at higher metals/metalloids concentrations (100.15 mg/kg), the suppressive effect of pH on bioaccumulation was attenuated.

## 4 Discussions

### 4.1 Divergent accumulation and translocation patterns among metals/metalloids

Cd is among the most extensively investigated heavy metal in the field of phytoremediation. Among the 39 known Cd hyperaccumulator species, *Sedum plumbizincicola* demonstrates the highest capacity for both Cd accumulation and translocation. This species shows enhanced Cd tolerance and accumulation, likely due to the activity of the *SaMT2* gene, which promotes Cd chelation, thus reducing the concentration of free Cd ions in plant cells (Zhang et al., 2014b). Furthermore, *SaMT2* acts as an antioxidant, mitigating reactive oxygen species generated by metal stress (Hassinen et al., 2011). The highest average BF for Cd is 10, whereas the average TF is markedly lower, at 1.8 ± 0.1 (**Figure 1A**).

Root-to-shoot translocation of metals/metalloids is often constrained by physiological mechanisms such as limited xylem loading and vacuolar compartmentalization, which restrict Cd movement to aerial tissues (Yang et al., 2021). Excessive Cd is stored in vacuoles, reducing its cytoplasmic concentration and minimizing organelle toxicity (Sharma et al., 2016). Prolonged stress or high Cd concentrations can weaken this root barrier, facilitating Cd transport to aerial parts (Yu et al., 2021). This mechanism is supported by our observation of a significantly positive correlation between Cd remediation duration and TF value (*p* < 0.001) (**Figure 7**). Furthermore, a significant negative correlation between AGB and BF (*p* < 0.001), alongside a positive correlation between AGB and TF (*p* < 0.001), suggests that increasing biomass leads to dilution of accumulated Cd due to enhanced allocation of energy to growth, thereby reducing its concentration per unit of biomass (Huang et al., 2020c). This indicates that Cd hyperaccumulators have strong root accumulation capacity, but lower TF due to relatively low AGB.

**Figure 8:**
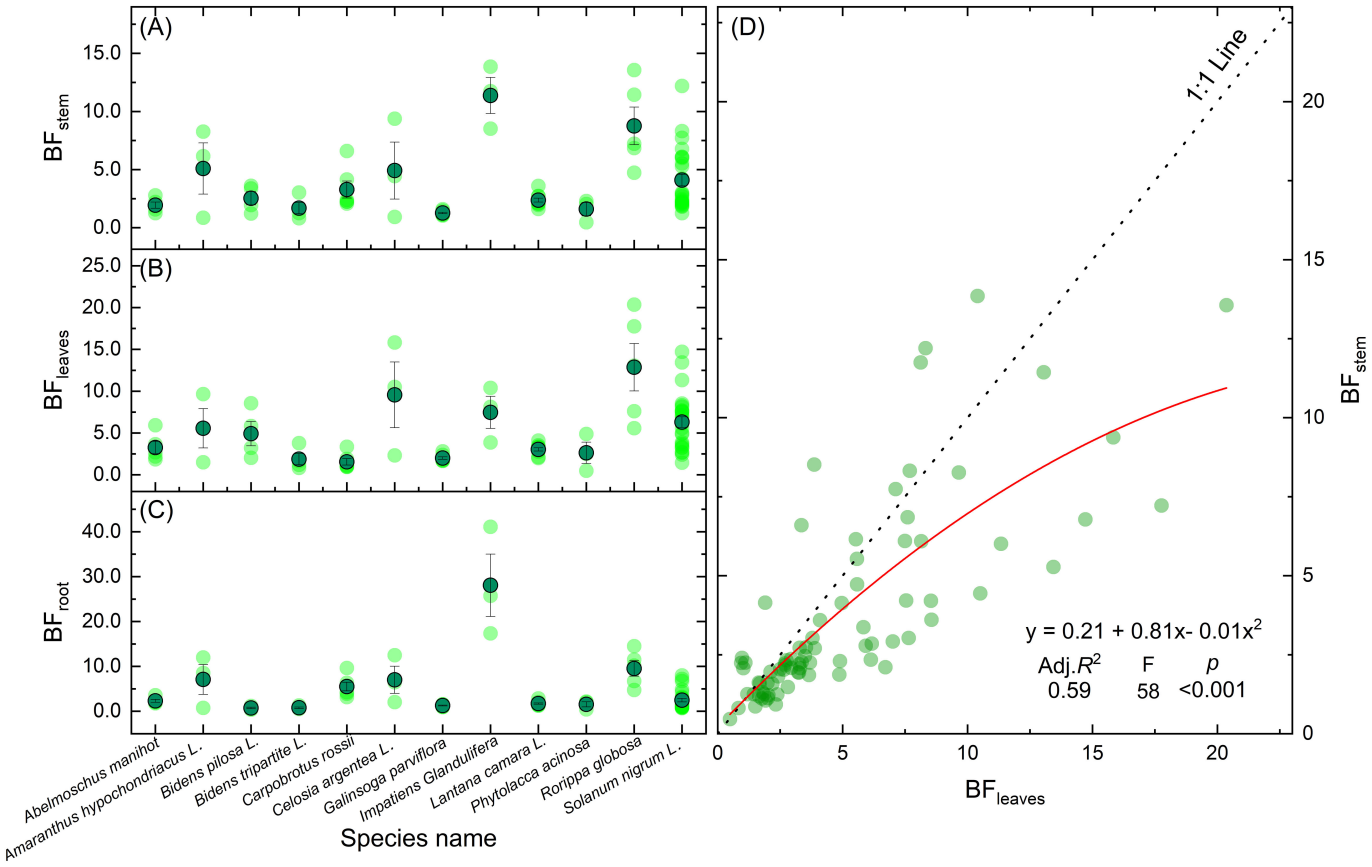
BFs of Cd in the stems **(A)**, leaves **(B)**, and roots **(C)** of 12 plant species and relationship between BF_leaves_ and BF_stem_ **(D)**.

Approximately 83% of hyperaccumulator plants translocate more Cd to leaves than stems (**Figure 8D**). As primary sites of photosynthesis, leaves possess high affinities for both water and minerals, making them key reservoirs for metals such as Cd via transpiration-driven transport (Song et al., 2017). Within leaf tissues, Cd toxicity is alleviated through complexation with organic acids (Hassan and Aarts, 2011) or chelation (Yadav, 2010), reinforcing the role of leaves as principal sinks for Cd. *Bidens pilosa* L., a notable Cd hyperaccumulator from the Asteraceae family, thrives in contaminated areas due to its high biomass, strong adaptability, and rapid growth. c (Sun et al., 2009a). Some hyperaccumulator species also show substantial Cd accumulation in stems. For instance, *Impatiens glandulifera* exhibits a stem BF ranging from 8.5 to 13.6, compared to a leaf BF of 3.9 to 10.4 (**Table 1**), indicating efficient root-to-stem translocation and Cd compartmentalization within branch cell walls to mitigate toxicity (Lai, 2014).

**Table 1:** BF of Cd in the stem, leaves, and roots of hyperaccumulating plants.

Species name	BF stem	BF leaves	BF roots	Ref.
*Abelmoschus manihot*	1.26~2.78	1.85~5.92	1.78~3.6	(Wu et al., 2018)
*Alopecurus aequalis*	–	–	10.26	(Ren et al., 2021)
*Alternanthera philoxeroide*	–	–	4.98	(Hu et al., 2013)
*Amaranthus hypochondriacus L.*	0.47~8.27	1.4~15.56	0.76~11.98	(Long et al., 2019; Huang et al., 2020)
*Bidens pilosa L.*	1.22~3.61	2.03~8.55	0.37~1.18	(Wei et al., 2008)
*Bidens tripartite L.*	0.82~3.03	0.83~3.79	0.51~1.29	(Wei et al., 2009)
*Carpobrotus rossii*	2.08~6.6	0.94~3.35	3.17~9.65	(Zhang et al., 2014a)
*Celosia argentea L.*	0.63~9.38	2.32~23.65	2.06~12.51	(Long et al., 2019; Huang et al., 2020)
*Chromolaena odorata*	–	–	1.77~6.49	(Wei et al., 2018)
*Coronopus didymus L.*	–	–	2.17~3.84	(Sidhu et al., 2017)
*Corydalis petrophila Franch*	–	–	0.16	(Yanqun et al., 2005)
*Corydalis pterygopetala Hand-Mazz*	–	–	0.26	(Yanqun et al., 2005)
*Crassocephalum crepidioides (Benth.) S. Moore*	18.05	24.83	9.03	(Zhu et al., 2018)
*Cynoglossum furcatum*	–	–	0.38	(Ren et al., 2021)
*Galinsoga parviflora*	1.05~1.6	1.66~2.8	0.95~1.58	(Lin et al., 2014)
*Impatiens Glandulifera*	8.52~13.85	3.86~10.4	17.36~41.1	(Coakley et al., 2019)
*Lantana camara L.*	1.61~3.59	1.99~4.09	0.53~2.86	(Liu et al., 2019)
*Lysimachia deltoides*	–	–	0.11	(Wang et al., 2009)
*Phytolacca acinosa*	0.11~11.03	0.49~44.68	0.43~2.18	(Nie, 2006; Long et al., 2019; Huang et al., 2020; Ren et al., 2021)
*Picris hieracioides L.*	–	–	0.38	(Yanqun et al., 2005)
*Plantage erosa Wall. In Roxb*	–	–	0.53	(Yanqun et al., 2005)
*Potentilla fulgens Wall.*	–	–	0.39	(Yanqun et al., 2005)
*Pterocypsela laciniata (Houtt.) C.*	–	–	1.4~2.08	(Zhong et al., 2019)
*Rorippa globosa*	4.73~13.56	5.58~20.36	4.74~14.52	(Wei and Twardowska, 2013)
*Sedum plumbizincicola*	–	28.37~58.67	–	(Huang et al., 2020)
*Silene viscidula*	–	–	0.54~0.54	(Wang et al., 2009)
*Solanum nigrum L.*	0.61~12.2	1.16~14.71	–	(Wei et al., 2005; Sun et al., 2008, Sun et al., 2009b; Han et al., 2016; Long et al., 2019; Huang et al., 2020)
*Youngia erythrocarpa*	–	–	2.4~2.92	(Lin et al., 2015)

**Figure 9:**
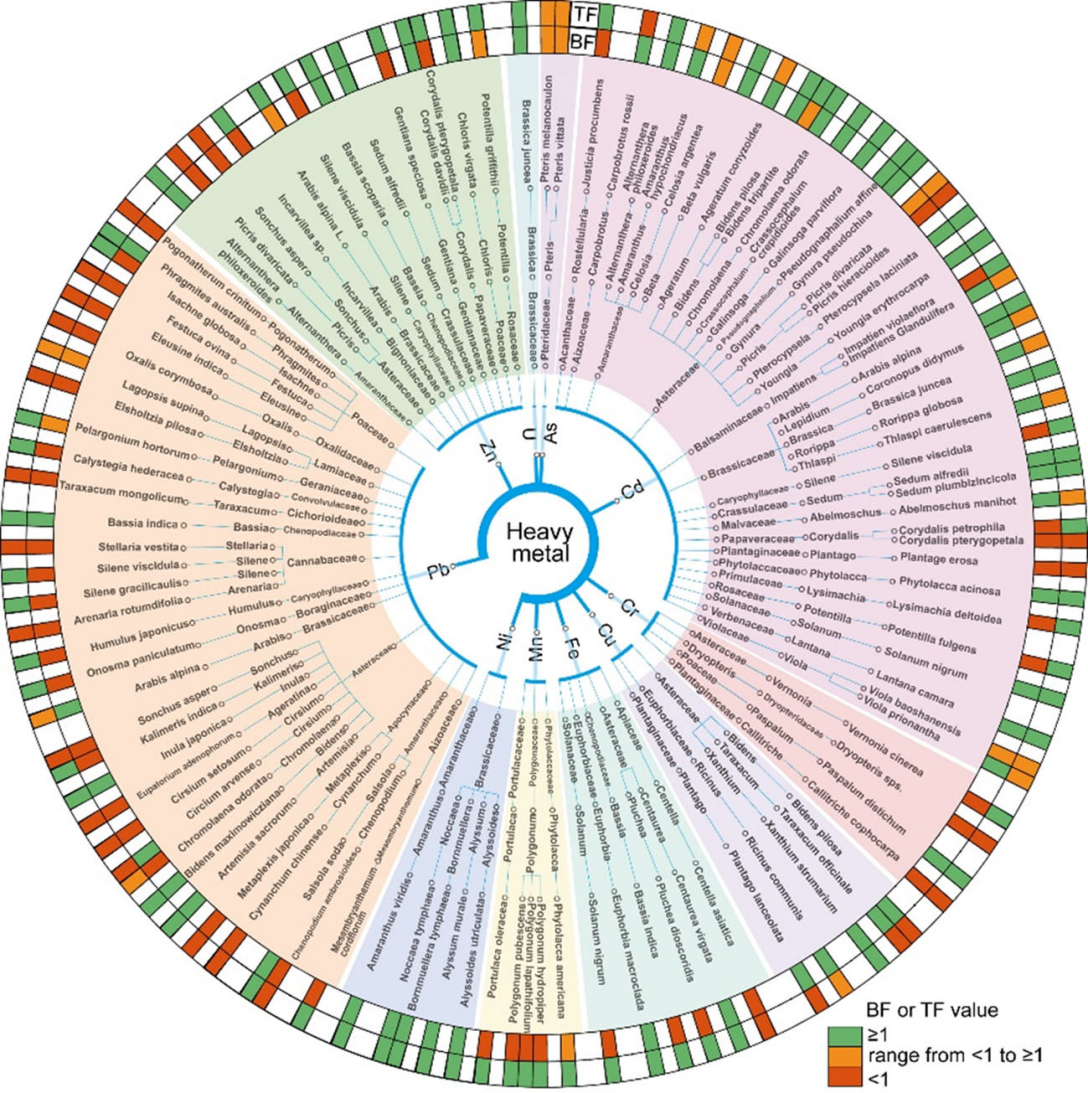
Classification map of hyperaccumulator plants. The hyperaccumulators of different heavy metal types were classified according to family, genus and species, and different colors were used to indicate the range of BF and TF value of each plant. Green means greater than 1, red means less than 1, and yellow means both greater than 1 and less than 1.

In contrast to Cd, Ni hyperaccumulators exhibit the highest average TF values, indicating that Ni is predominantly accumulated in the AGB rather than in the roots. Our results indicate that most Ni hyperaccumulators belong to the Brassicaceae and Amaranthaceae families (**Figure 9**). Among these, *Noccaea tymphaea* demonstrates exceptional translocation capacity (TF=135.5), with Ni complexed by low-molecular-weight carboxylic acids in leaves (Chardot et al., 2005). *Bornmuellera tymphaea* (TF=118.3) shows Ni-availability dependent accumulation, while *Alyssum murale* (TF=15.1) absorption correlates with soil Ni and transpiration rate (Centofanti et al., 2012). The serpentine-adapted *Alyssoides utriculata* (TF=4.7) can accumulate over 1000 μg g^-^¹ Ni and exhibits an inverse Ca/Mg-Ni relationship, likely due to competitive ion uptake mechanisms (Van Der Ent et al., 2013; Proctor and Mcgowan, 1976).

Limited data also indicate contrasting patterns. For instance, Cr hyperaccumulators (e.g., *Salsola kali*) exhibit higher root retention (average TF = 0.8) despite moderate BF (~3.3), likely due to Cr(III) precipitation in root vacuoles (Kramer, 2010). Similarly, As exhibits intermediate translocation (TF = 4.2) but strong leaf sequestration in Pteris vittata, where As(V) is reduced to As(III) and subsequently complexed with thiol-containing compounds (Zhao et al., 2009). Fe, despite its low average BF (0.8), may achieve efficient translocation (TF>2) under flooded soil conditions, facilitated by phytosiderophore-mediated uptake, as observed in *Oryza sativa* (Morrissey and Guerinot, 2009).

### 4.2 pH-dependent metals/metalloids bioavailability

Soil pH is one of the most critical factors affecting the transformation of metals from non-fixed solid phase to a bioavailable soluble forms (Sun et al., 2016). Our analysis reveals that BFs for Cd and Zn decrease with increasing soil pH. In low pH soils, the solubility of Cd and Zn is enhanced, thereby facilitating their uptake by plants (Mossa et al., 2021), a trend confirmed by our findings (**Figures 4**, **6**). Acidic conditions increase proton (H^+^) concentrations, which compete with metal ions for adsorption sites, releasing more metal ions into the soil solution. Additionally, soil organic matter binds metals more effectively in acidic conditions, further enhancing solubility (Jones et al., 2003). Soils with low pH also have weaker buffering capacities, increasing the mobility and bioavailability of metals (Najafi and Jalali, 2016). Interestingly, Pb exhibits an opposite trend, with BFs increasing as soil pH rises. This could be due to the increased solubility of Pb in alkaline conditions, as confirmed by a significant negative correlation between available soil metals and bioaccumulation factors (*p* < 0.001) (**Figure 5**). Unlike Cd and Zn, Pb does not form insoluble compounds in high pH conditions. Cu and Zn, however, tend to form insoluble hydroxides in alkaline conditions, reducing their bioavailability. Notably, Cr and As bioavailability exhibits pH-dependence distinct from Cd/Pb: Cr(VI) mobility peaks at neutral pH (6-8), while As(V) adsorption increases with Fe/Mn oxide content in acidic soils (Morrissey and Guerinot, 2009). This explains why As hyperaccumulators (e.g., *Pteris vittata*) perform optimally in slightly acidic soils (pH 5.5-6.5), whereas Cr-tolerant species (e.g., *Calluna vulgaris*) thrive in alkaline conditions where Cr(III) dominates (Mayes et al., 2005).

### 4.3 Metal/metalloids toxicity thresholds in hyperaccumulating plants

Due to the complex chemical speciation of metals in soil, total metal/metalloid concentrations are often poor predictors of their bioavailability to plants (Chen et al., 1996). Our results show a significant negative correlation between the B) and total soil metal/metalloid content (*R*² = 0.49, *p* < 0.001), with BF decreasing as concentrations rise (**Figure 5A**). When soil metal/metalloid levels exceed 1000 ppm, BF typically drops below 1, likely due to toxicity constraints on plant growth and uptake. **Figure 5B** further illustrates metal-specific thresholds: for instance, Cd concentrations exceeding 201 ppm result in BF values below 1, while Zn requires concentrations over 21,000 ppm to reach similar reductions. This suggests that plants accumulate Cd less efficiently than Zn at lower concentrations, with Zn being essential for plant processes. As a result, plants can better accumulate Zn at higher concentrations, whereas Cd’s toxicity limits its uptake and transport. Simultaneously, as bioavailable metal/metalloid concentrations increase, plant accumulation capacity decreases significantly (*p* < 0.001) (**Figure 6**). Additionally, when available Cd exceeds 100 ppm, BF drops below 1 (**Figure 6A**). This aligns with previous studies showing that higher total metal concentrations lead to increased bioavailability (Parizanganeh et al., 2010), but plant accumulation is limited by a toxicity threshold. Once metal levels exceed a critical point, their toxicity impairs plant growth, further limiting accumulation (Baker et al., 1994).

### 4.4 Environmental and temporal drivers of metal/metalloids bioaccumulation

The efficiency of plants in accumulating and translocating metals/metalloids varies with experimental conditions, with BF and TF values generally higher in historically contaminated soils compared to newly contaminated soils (**Figure 2**). This findings indicate that long-term exposure enables plants at historically contaminated sites to evolve enhanced uptake and translocation mechanisms, achieving metal stabilization through chelator biosynthesis and precise regulation of transporter protein activity (Clemens, 2001; Andrés-Colás et al., 2006; Chi et al., 2019; Anjitha et al., 2021; Zheng et al., 2023). Additionally, plants enhance their antioxidant systems to combat metal-induced oxidative stress. In contrast, plants in newly contaminated soils may lack these adaptations, leading to lower accumulation efficiency. Rhizosphere microorganisms, such as mycorrhizal fungi and bacteria, also aid metal uptake by altering pH, secreting chelators, and promoting root growth, with microbial resistance mechanisms playing a critical role in plant survival and metal uptake (Abdu et al., 2017). Additionally, BF and TF values for Cd, Zn, Ni, and Pb are much higher in laboratory conditions than in the field (**Figure 3**). This difference is due to variations in plant physiology, soil properties, and climate. Laboratory conditions provide stable temperature, moisture, and nutrients, while field conditions are subject to environmental fluctuations, such as extreme weather, which can negatively affect plant growth and reduce metal accumulation efficiency (Hatfield and Prueger, 2015).

Climatic characteristics, remediation duration, soil properties, AGB, and plant characteristics strongly influenced the BF and TF of Cd hyperaccumulators. Our results indicate a positive correlation between BF and MAT (*p* < 0.001). The average BF values at MAT intervals of <10°C, 10–15°C, 15–20°C, and >20°C were 4.06, 1.43, 5.52, and 8.43, respectively. Higher MAT promotes metabolic rates and enhances root activity, resulting in increased contaminant uptake (Chang et al., 2015). Additionally, temperature indirectly regulates metals/metalloids bioavailability by affecting soil moisture and microbial activity. This may explain why plants grown under laboratory conditions often show greater metals/metalloids accumulation than those grown in field conditions.

The BF and TF values of Cd hyperaccumulators were observed to increase with prolonged remediation duration. This suggests that extended exposure improves the tolerance and adaptability of hyperaccumulators to Cd, allowing them to thrive in Cd-contaminated soils. Initially, plants respond to metal stress by activating antioxidant enzyme systems and synthesizing specific amino acids (Kardos et al., 2018). The response time to metals/metalloids stress varies from hours to days or even longer, depending on the severity of the stress and the plant’s adaptive mechanisms (Ogawa et al., 2009). As shown in **Figure 7 a** significant positive correlation exists between plant accumulation and translocation capacities and the duration of exposure. This indicates that adaptation to heavy metal stress is a dynamic process, involving various physiological and molecular adjustments. Although this adaptation occurs gradually over time, the exact duration required remains unclear in current research.

### 4.5 Species selection guidelines for practical phytoremediation

This study provides key insights for the formulation of standardized phytoremediation protocols targeting metal-contaminated soils. Plant selection should be tailored to the specific metal/metalloid profiles of contaminated sites, based on species-specific accumulation and translocation capacities (**Figure 9**). Hyperaccumulator species such as *Sedum plumbizincicola* are effective candidates for the phytoremediation of Cd- and Zn-contaminated soils. In Pb-contaminated sites, *Bassia indica* serves as an effective species for *in situ* stabilization, reducing Pb mobility through root-associated immobilization processes. For Ni or Cr contaminated sites, hyperaccumulator species such as *Alyssum murale* and *Paspalum distichum* demonstrate strong accumulation and translocation capabilities, making them suitable candidates for phytoremediation. These species-specific strategies provide a targeted and practical approach to remediating diverse types of heavy metal and metalloid contamination under field conditions. Maintaining acidic soil conditions (pH < 6) significantly enhances the phytoremediation efficiency for Cd and Zn, whereas slight alkalization (pH 6–7) improves Pb uptake. These findings provide a practical foundation for incorporating soil pH management into phytoremediation strategies.

## 5 Conclusions

This study systematically evaluated the bioaccumulation and translocation capacities of hyperaccumulator plants for various metals/metalloids (e.g., Cd, Ni, Zn, Pb) through 547 published observations. The results revealed pronounced metal-specific differences in accumulation and transport strategies—most notably, the contrasting behavior of Cd (high BF, low TF) versus Ni (high TF). Soil pH emerged as a key environmental driver influencing these patterns by modulating metal speciation and availability. Threshold concentrations (e.g., ~214.8, 669.5, 31352.3, 8291.1, and 18657.3 mg kg^-^¹ for Cd, Cu, Pb, As, and Zn, respectively) were identified as critical tipping points beyond which metal toxicity constrains the bioaccumulation capacity of hyperaccumulators. These insights underscore the importance of matching metal-specific uptake traits with contamination profiles and leveraging soil properties to optimize phytoremediation. Collectively, this study offers a mechanistic and practical framework for advancing phytoremediation strategies under diverse soil conditions.

## Supplementary material

Supplementary Figure 1 Funnel plot of combined BF for heavy metal uptake in plant across different soil conditions. The vertical dotted line indicates the mean effect size, while the outer dotted lines represent approximate 95% confidence limits.

Supplementary Figure 2 Funnel plot of combined TF for heavy metal uptake in plant across different soil conditions. The vertical dotted line indicates the mean effect size, while the outer dotted lines represent approximate 95% confidence limits.

Supplementary Figure 3 Leave-one-out sensitivity analysis of combined effect sizes (log-transformed BF and TF). Each point represents the recalculated pooled effect size after omitting one observation at a time. The red dashed line indicates the overall mean effect size across all observations.

Supplementary Figure 4 Model diagnostics for the log(BF) mixed model.

Supplementary Figure 5 Interaction effect of soil pH and total metal content on log(BF). Predicted values of log(BF) were derived from a linear mixed-effects model, with standardized soil pH and total metal content included as fixed effects, and plant species as a random intercept. Shaded areas indicate 95% confidence intervals. Metal content levels were categorized into three representative values corresponding to the 25th percentile (low: 3.39 mg/kg), 50th percentile (medium: 10.74 mg/kg), and 75th percentile (high: 100.15 mg/kg) of total metal concentrations.
