# Supplementary figures and images for: Systematic evaluation of plant metals/metalloids accumulation efficiency: a global synthesis of bioaccumulation and translocation factors
Source: Front Plant Sci. 2025 Jun 5;16:1602951. doi: 10.3389/fpls.2025.1602951 (PMC12178126; doi:10.3389/fpls.2025.1602951)

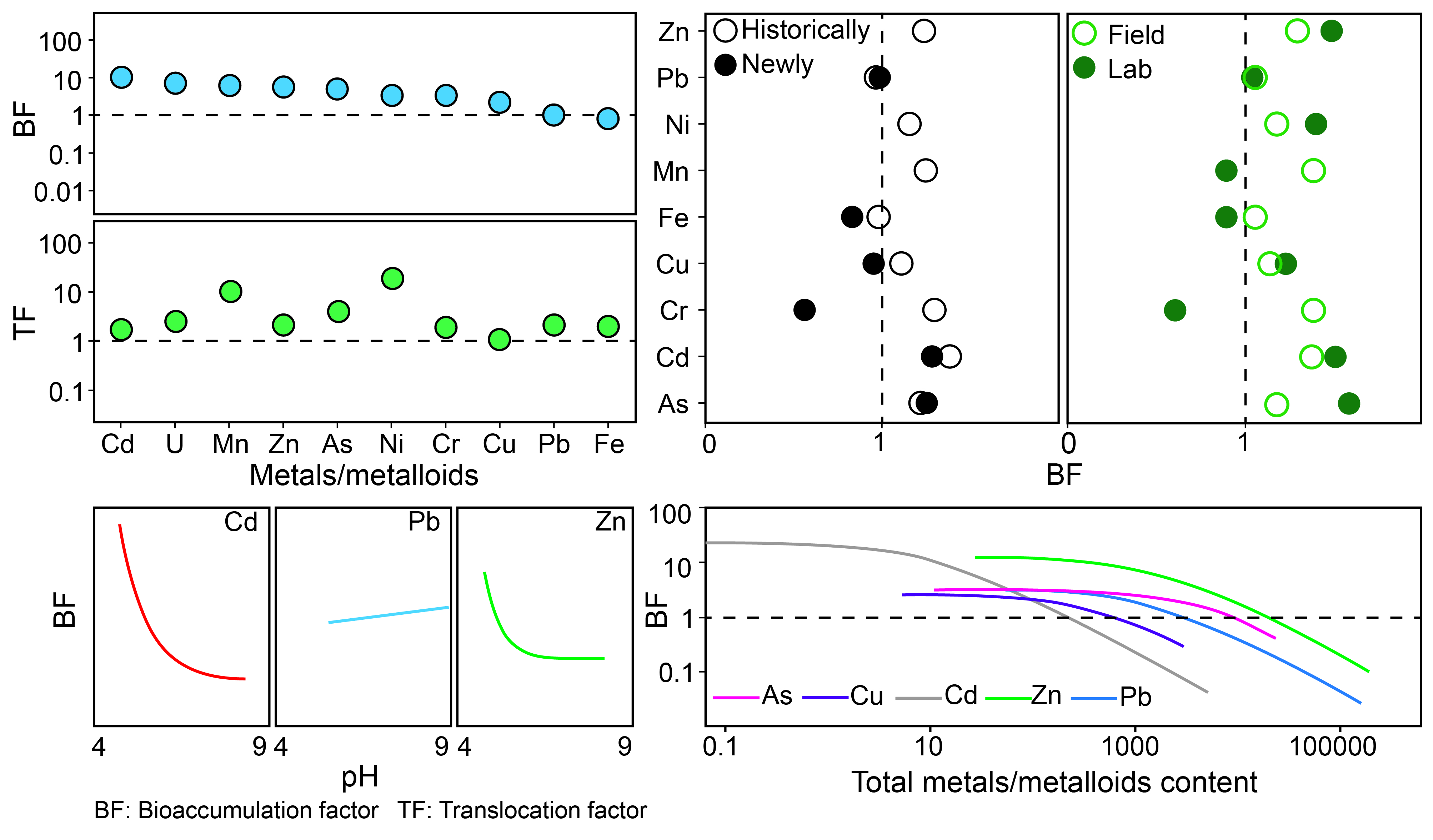

Supplement: Supplementary Figure 1 — Funnel plot of combined BF for heavy metal uptake in plant across different soil conditions. The vertical dotted line indicates the mean effect size, while the outer dotted lines represent approximate 95% confidence limits. [file Image1.jpeg]

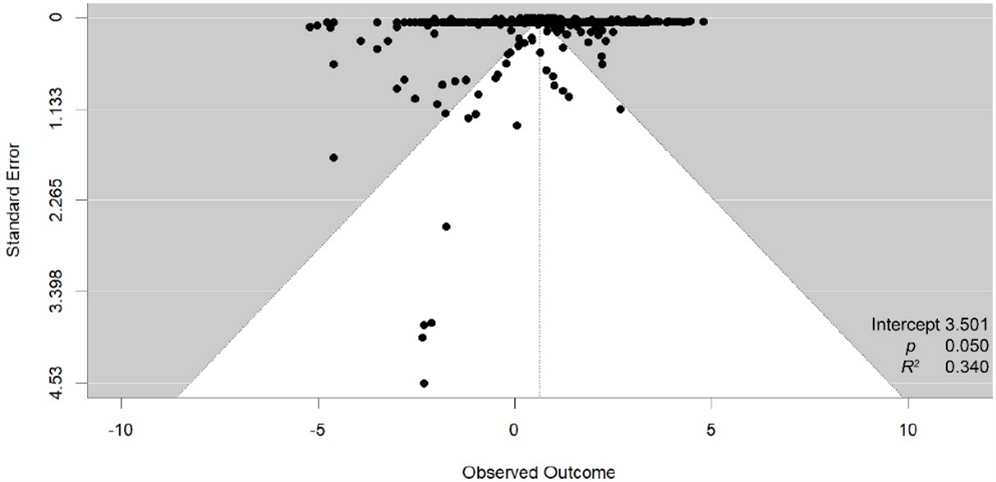

Supplement: Supplementary Figure 2 — Funnel plot of combined TF for heavy metal uptake in plant across different soil conditions. The vertical dotted line indicates the mean effect size, while the outer dotted lines represent approximate 95% confidence limits. [file Image2.tif]

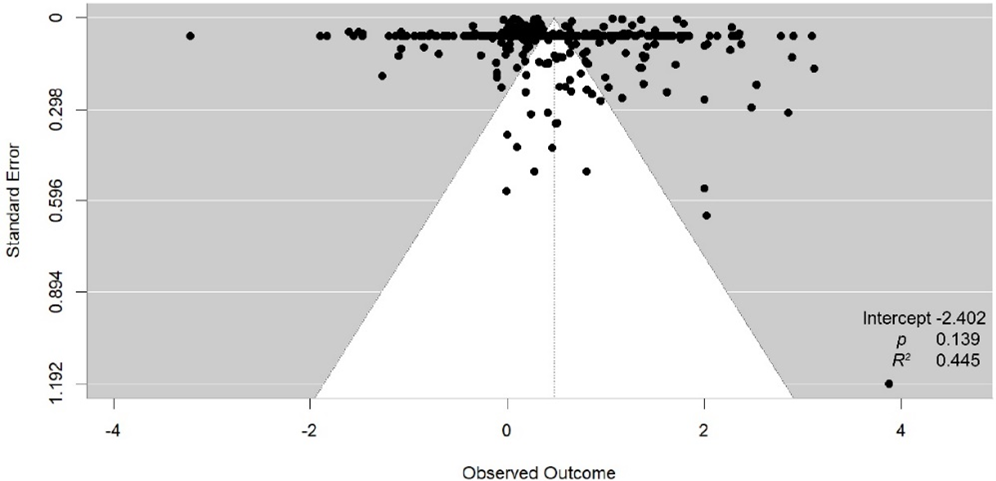

Supplement: Supplementary Figure 3 — Leave-one-out sensitivity analysis of combined effect sizes (log-transformed BF and TF). Each point represents the recalculated pooled effect size after omitting one observation at a time. The red dashed line indicates the overall mean effect size across all observations. [file Image3.tif]

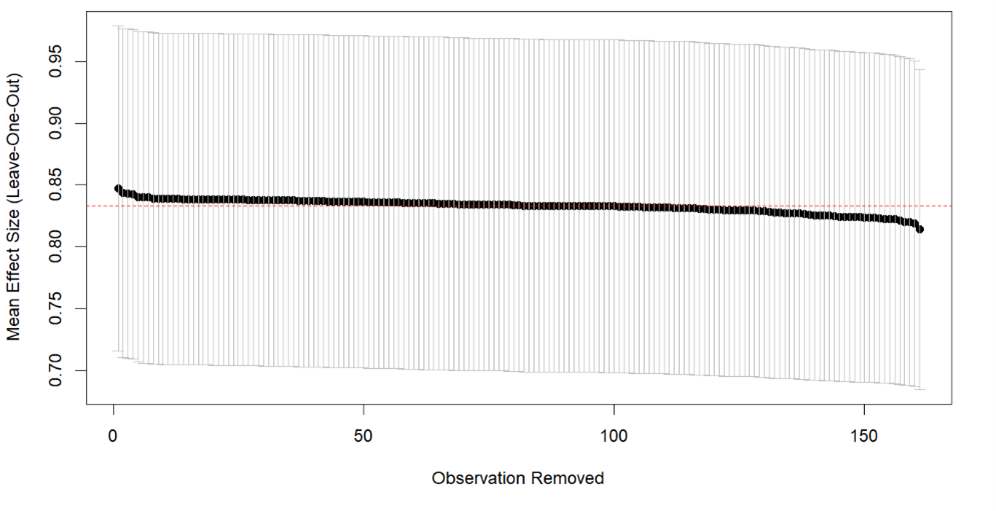

Supplement: Supplementary Figure 4 — Model diagnostics for the log(BF) mixed model. [file Image4.tif]

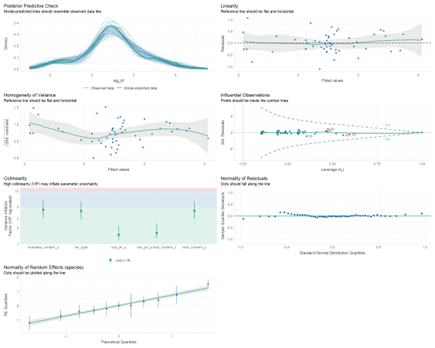

Supplement: Supplementary Figure 5 — Interaction effect of soil pH and total metal content on log(BF). Predicted values of log(BF) were derived from a linear mixed-effects model, with standardized soil pH and total metal content included as fixed effects, and plant species as a random intercept. Shaded areas indicate 95% confidence intervals. Metal content levels were categorized into three representative values corresponding to the 25th percentile (low: 3.39 mg/kg), 50th percentile (medium: 10.74 mg/kg), and 75th percentile (high: 100.15 mg/kg) of total metal concentrations. [file Image5.tif]

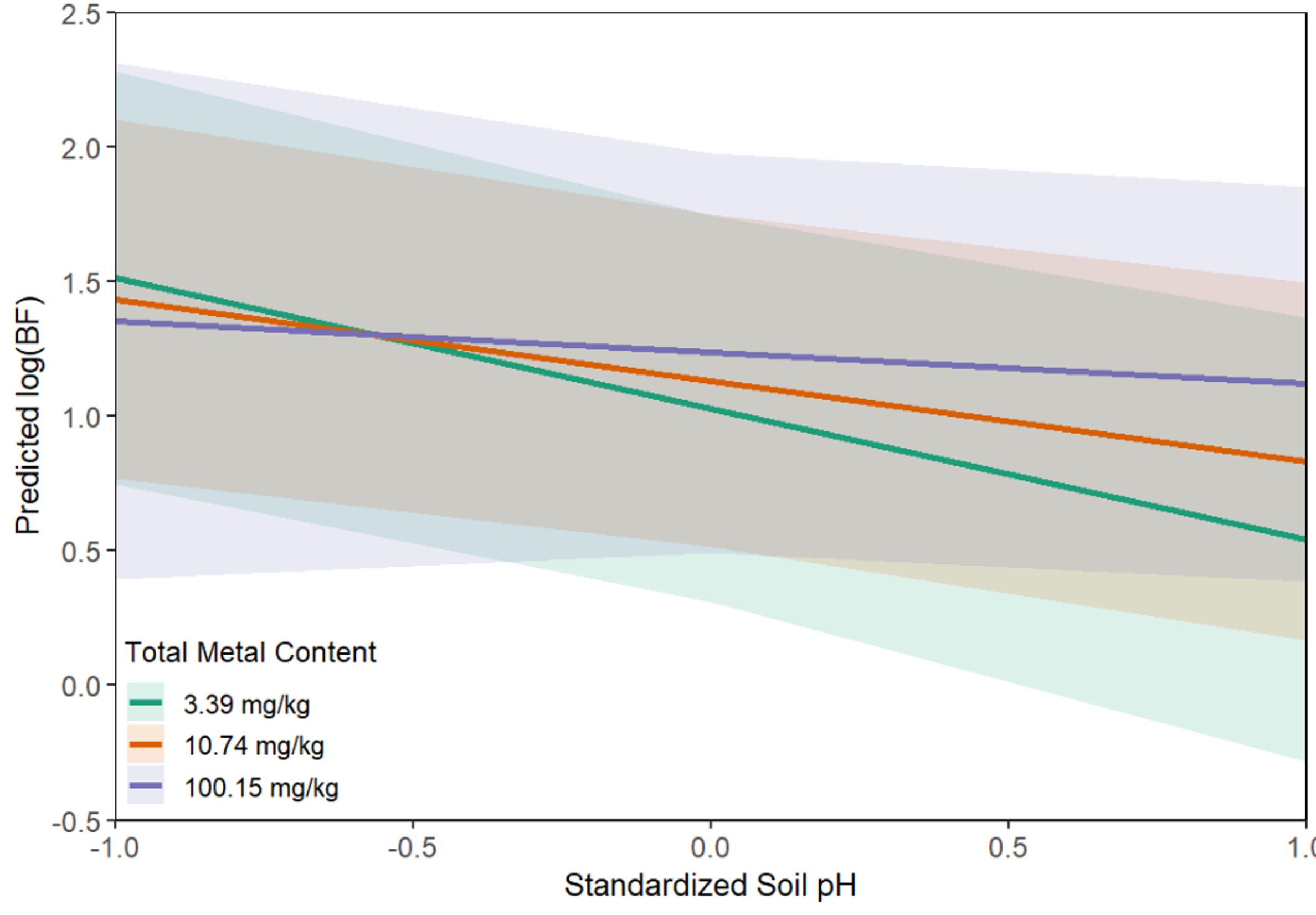

Supplement: Supplementary file 6 [file Image6.tif]
